# *In vitro* assessment of thermal changes during piezosurgery and rotary osteotomy

**DOI:** 10.6026/973206300214818

**Published:** 2025-12-15

**Authors:** Ipsita Jayanti, Ataul Hafeez Imran, Aditya Narayan Shukla, Vishwannath Hiremath, Sourav Sen, Spandan Barui

**Affiliations:** 1 Department of Periodontology, Dr. G.D.Pol Foundation YMT Dental College and Hospital, Kharghar, Navi Mumbai; 2 Department of Dental Surgery, FICOI(USA), AFLD (WCLI,USA) Noor Hospital, Qadian ,District Gurdaspur, India; 3 Department of Oral & Maxillofacial Surgery, Babu Banarasi Das College of Dental Sciences, Lucknow, Uttar Pradesh, India; 4 Department of Oral and Maxillofacial Surgeon, Dr Hiremath Hospitals (A Unit of Hiremath Hospitals Pvt Ltd) Vijayanagar Bangalore, India; 5 Department of Public Health Dentistry, Maharishi Markandeshwar College of Dental Sciences and Research, Mullana, Ambala, 133207; 6 Department of Pediatric And Preventive Dentistry, Kalinga Institute Of Dental Sciences, KIIT Deemed To Be University, Bhubaneswar, Odisha, India

**Keywords:** Piezosurgery, osteotomy, thermal injury, bone temperature, rotary instruments, ultrasonic surgery, bone necrosis, thermometry

## Abstract

High temperatures in osteotomy may result in thermal bone necrosis, which would compromise the healing process and the process of osseointegration. Hence, temperature increase in bone during osteotomy with piezosurgery and conventional rotary tools in different irrigation conditions were compared. Sixty bovine bone samples were examined with the help of thermocouples to define the real time temperature changes. Even with no irrigation, piezosurgery produced much lower peak temperatures and slower rates of temperature rise than was produced with rotary instruments. Thus, we show the claim that piezosurgery is safer in terms of thermal and less prone to bone injuries caused by heating during osteotomy.

## Background:

Osteotomy operations are one of the basic elements of oral and maxillofacial surgery, orthopedics and dental implants installation [1]. Bone cutting heat is an inevitable effect of the mechanical conversion of energy and there is a risk of thermal damage to the osseous tissues when the temperature levels are surpassed [2]. Both experimental and clinical studies have shown that bone temperatures above 47C over a period of time longer than one minute cause permanent thermal necrosis, which is the death of osteocytes, the denaturation of proteins and impaired bone regeneration [3]. The traditional rotary tools such as high speed drills and saws have been used as the methodology to prepare bones decades ago [4]. These devices rotate at a rate of 20,000 to 40,000 rpm and their cutting is through the use of mechanical friction against the mineralized tissue. Nevertheless, heat production through friction especially where ineffective cooling or irrigation is used poses a great danger to thermal bone damage [5]. Delays in healing, suture fibrosis, intracortical encapsulation of tissue and implant failure are some of the sequelae of necrosis in dental applications that are caused by heat [6]. Piezosurgery is a recent technology that was introduced in the early 2000s and uses ultrasonic vibrations of a frequency of 25-29 kHz with micrometric oscillations (60-200 um ) to selectively cut hard tissues and leave soft tissues intact [7]. Theoretical benefits encompass low heat production, low surgical errors, better visibility by cavitation effect and cut selectivity, which causes minimal effects on the soft tissue [8]. Theoretical benefits of the micromechanical cutting action include a more efficient distribution of energy compared to the macroscopically rotary friction and, therefore, the thermal elevation may be reduced [9]. Although the use of thermal safety piezosurgery compared to conventional rotary instruments in clinics is on the rise, comparative statistics are still inconsistent [10]. Some of the studies have indicated reduced temperature production using ultrasonic equipment and other studies have indicated similar or even higher temperatures based on the operating parameters such as power levels, tip shape and irrigation procedures [11]. Several factors among the investigations prove to be difficult in direct comparisons and clinical extrapolation due to methodological differences such as the various temperature measuring methods, bone model and experimental designs [12]. There still exist critical knowledge gaps in real time dynamics of temperatures during osteotomy procedures, spatial parameters of thermal variations at different distances of cutting site, influences of various irrigation regimes on temperature control and duration of temperature rise above critical level [13]. Attempts to locate single-point temperatures or infrared thermography with a low spatial resolution of temperatures, most of the past studies have not been able to provide a three-dimensional thermal gradient in bone [14]. Moreover, there are no literature sources that can provide standardized *in vitro* models that allow comparing piezosurgery and rotary instruments under the same conditions [15]. This type of comparative information is vital to the evidence-based choice of instruments, the creation of surgical practices that reduce the risk of thermal injury and the efficiency of cooling procedures in bone surgery. Therefore, it is of interest to make a detailed comparison of thermal changes in bone when performing a standardized osteotomy with the use of piezosurgery and traditional rotary tools. The following objectives were set: (1) to measure the maximum temperature rise and rates of increase of temperature; (2) to measure the spatial thermal distribution at various distances per osteotomy site; (3) to evaluate the impact of various irrigation protocols on thermal regulation; (4) to obtain the duration of the increase of temperature beyond the critical values; and (5) to acquire the risk factors of surpassing safe thermal limits. We postulated that piezosurgery would produce substantially low temperatures than rotary instruments in all the conditions we tested.

## Materials and Methods:

## Study design and ethical considerations:

This comparative *in vitro* experimental study was conducted at the Oral and Maxillofacial Surgery between February 2024 and October 2024

## Sample selection and preparation:

## Bone specimen procurement: 

Fresh bovine rib bones were obtained from a local abattoir within 6 hours of animal sacrifice. Ribs were selected due to their cortical-cancellous bone composition similar to human jaw bone, established use in bone cutting research and standardized geometry facilitating reproducible experimentation.

## Inclusion criteria:

Fresh bovine ribs from animals aged 18-24 months; intact cortical bone without fractures or defects; bone thickness 8-12mm; absence of visible pathology.

## Exclusion criteria:

Bones with visible cracks, erosions, or pathological changes; specimens with irregular geometry preventing standardized osteotomy; bones stored >12 hours post-harvest; evidence of previous freezing.

## Sample size calculation:

Based on pilot data showing mean temperature difference of 4.5°C between groups with pooled standard deviation of 3.2°C, assuming α=0.05 and power=0.90, minimum sample size of 22 per group was calculated. To account for potential technical failures and enable subgroup analyses, 30 specimens per group (total n=60) were included.

## Specimen preparation:

Ribs were cleaned of soft tissue, sectioned into 50mm segments using a precision saw under copious water cooling and stored in physiological saline (0.9% NaCl) at 4°C for maximum 24 hours before testing. Immediately prior to experimentation, specimens were equilibrated to room temperature (23 ± 1°C) for 2 hours. Bone mineral density was measured using dual-energy X-ray absorptiometry (DEXA) to ensure comparability between groups.

## Experimental groups and randomization:

Specimens were randomly allocated using computer-generated random numbers to two main groups:

Group 1 - Piezosurgery (PS, n=30): Osteotomies performed using Piezosurgery device (Mectron Piezosurgery 3, Mectron Medical Technology, Italy) with standardized bone cutting insert (OT7S, cutting tip width 2mm), frequency 25-29 kHz, power setting "Medium" (corresponding to approximately 60W), amplitude modulation "boost mode" activated.

Group 2 - Rotary Instruments (RI, n=30): Osteotomies performed using electric high-speed handpiece (W & H Alegra, W & H Dentalwerk, Austria) with cylindrical carbide bur (diameter 2mm, length 10mm, cross-cut design), rotational speed 40,000 rpm, torque 60 Ncm.

Each main group was further subdivided into three irrigation protocol subgroups (n=10 each):

[1] Subgroup A: Continuous external irrigation (25ml/min sterile saline, 20°C)

[2] Subgroup B: Intermittent irrigation (5ml/min, applied every 5 seconds)

[3] Subgroup C: No irrigation (dry cutting)

## Osteotomy protocol:

Standardized osteotomies were created following strict protocol:

[1] Dimensions: 10mm length x 3mm depth x 2mm width

[2] Orientation: Perpendicular to bone surface

[3] Location: Center of prepared bone segment

[4] Technique: Single continuous pass for rotary instruments; multiple overlapping passes for piezosurgery (as per manufacturer recommendations)

[5] Pressure: Standardized using force-sensing platform maintaining constant 2N applied force

[6] Operator: All procedures performed by single experienced operator (>5 years surgical experience) to eliminate inter-operator variability

## Temperature measurement system:

## Thermocouple array:

Custom fabrication of K-type thermocouples (Type K, Omega Engineering, diameter 0.5mm, response time <0.1 second, accuracy ±0.5°C, temperature range -50 to +200°C) inserted into pre-drilled holes at precisely controlled distances from the planned osteotomy site: 1mm, 3mm and 5mm depth. Thermocouples were positioned at mid-depth of cortical bone (4-6mm from surface) using a precision drilling template.

## Data acquisition:

Real-time temperature monitoring employed multichannel data logger (Omega OM-DAQ-USB-2401, sampling rate 10 Hz) connected to laptop computer running proprietary software (Omega DaqView). Temperature recordings commenced 30 seconds before osteotomy initiation and continued for 120 seconds post-completion to capture temperature rise and dissipation phases.

## Baseline temperature:

Recorded for 30 seconds before each osteotomy; mean baseline temperature (T_baseline) was 23.2 ± 0.4°C across all specimens.

## Outcome measures:

## Primary outcomes:

[1] Maximum temperature achieved (T_max,°C)

[2] Temperature increase (ΔT = T_max - T_baseline,°C)

[3] Time to reach critical threshold of 47°C (seconds)

[4] Percentage of specimens exceeding 47°C

[5] Rate of temperature rise (°C/second during cutting phase)

## Secondary outcomes:

[1] Duration of temperature >43°C (threshold for reversible thermal injury)

[2] Temperature at procedure completion

[3] Time to return to baseline +5°C after procedure cessation

[4] Spatial temperature distribution (gradient analysis)

[5] Total procedure duration

## Quality control and standardization:

## Instrument maintenance:

New burs used for every 5 specimens; piezosurgery tips inspected and replaced if wear detected; handpieces calibrated weekly.

## Environmental control:

All experiments conducted in temperature-controlled room (23 ± 1°C, 50 ± 5% relative humidity).

## Irrigation standardization:

Calibrated peristaltic pumps delivered precise flow rates; irrigation temperature monitored (20 ± 1°C).

## Thermocouple validation:

Calibration against NIST-traceable reference thermometer before experiments; functional testing in water bath at multiple temperatures.

## Statistical analysis:

Statistical analyses were performed using SPSS version 28.0 (IBM Corporation) and GraphPad Prism version 9.0 (GraphPad Software). Descriptive statistics included mean ± standard deviation for continuous variables and frequencies (percentages) for categorical variables. Normal distribution was assessed using Shapiro-Wilk test and visual inspection of Q-Q plots. Homogeneity of variance was evaluated using Levene's test.

## Between-group comparisons:

[1] Independent t-tests compared continuous outcomes between piezosurgery and rotary instrument groups

[2] Two-way ANOVA examined effects of instrument type and irrigation protocol with interaction term

[3] Chi-square test compared proportions exceeding critical thresholds

## Within-group comparisons:

[1] Repeated measures ANOVA evaluated temperatures across multiple thermocouple positions

[2] Post-hoc pairwise comparisons employed Bonferroni correction

Pearson correlation assessed relationships between procedure duration, temperature elevation and bone density. Statistical significance was set at p<0.05 (two-tailed). Effect sizes were calculated using Cohen's d for continuous outcomes.

## Results:

All 60 specimens completed the experimental protocol without technical failures. Baseline characteristics were comparable between the piezosurgery (PS) and rotary instrument (RI) groups, with no statistically significant differences in bone mineral density (PS: 1.34 ± 0.11 g/cm^2^; RI: 1.36 ± 0.13 g/cm^2^; p = 0.523), cortical thickness (PS: 9.8 ± 1.2 mm; RI: 10.1 ± 1.4 mm; p = 0.387), or baseline temperature (PS: 23.1 ± 0.4°C; RI: 23.3 ± 0.5°C; p = 0.128). Mean osteotomy completion time was significantly longer for piezosurgery (47.3 ± 8.6 s) compared with rotary instruments (28.4 ± 5.2 s), representing a 66.5% increase in procedure duration (p < 0.001). As summarized in Table 1 (see PDF), maximum temperature (T_max), temperature increase from baseline (ΔT), and rate of temperature rise measured at 1 mm from the osteotomy site were consistently lower for piezosurgery than for rotary instruments under all irrigation conditions (all p < 0.001). Under continuous irrigation, neither instrument exceeded the critical threshold of 47°C. With intermittent irrigation, rotary instruments exceeded 47°C in 6 specimens (60%), whereas piezosurgery did not exceed this threshold in any specimen. Under no irrigation, rotary instruments exceeded 47°C in 8 specimens (80%), while piezosurgery again remained below this threshold. Two-way ANOVA demonstrated significant main effects of instrument type (F(1,54) = 287.4, p < 0.001) and irrigation protocol (F(2,54) = 124.6, p < 0.001), as well as a significant interaction between instrument type and irrigation protocol (F(2,54) = 18.3, p < 0.001), indicating that the magnitude of cooling differed between instruments across irrigation conditions. Spatial temperature distribution under continuous irrigation is presented in Table 2 (see PDF). Maximum temperature decreased significantly with increasing distance from the osteotomy site for both instruments (p < 0.001). Rotary instruments maintained significantly higher absolute temperatures than piezosurgery at all measured distances (1, 3, and 5 mm; all p ≤ 0.003). The temperature gradient between 3 and 5 mm was significantly steeper for rotary instruments compared with piezosurgery (3.10 ± 0.51 vs 2.40 ± 0.36°C/mm; p = 0.002), whereas the gradient between 1 and 3 mm did not differ significantly between instruments (p = 0.147). Temporal temperature characteristics are detailed in Table 3 (see PDF). Piezosurgery did not exceed 47°C under any irrigation condition, while rotary instruments exceeded this threshold under intermittent and no-irrigation conditions. The duration of temperature elevation above 43°C was significantly shorter for piezosurgery than for rotary instruments across all irrigation protocols (all p < 0.001). Time to peak temperature was significantly shorter for rotary instruments than for piezosurgery under all irrigation conditions (all p < 0.001). Recovery time to baseline + 5°C was significantly faster with piezosurgery compared to rotary instruments for continuous, intermittent, and no-irrigation protocols (all p ≤ 0.003). Correlation analysis showed a strong positive association between osteotomy duration and ΔT for rotary instruments (r = 0.742, p < 0.001), whereas the correlation was weaker and not statistically significant for piezosurgery (r = 0.284, p = 0.129). Bone mineral density demonstrated weak, non-significant inverse correlations with temperature elevation for both piezosurgery (r = -0.247, p = 0.189) and rotary instruments (r = -0.312, p = 0.094), indicating minimal influence within the studied range.

## Discussion:

Such an overall *in vitro* study shows that piezosurgery produces very low temperatures compared to traditional rotary tools during standardized osteotomy sessions in all the protocols tried, with piezo surgery holding temperatures below the critical 47C limit even in the absence of irrigation. These results give quantitative support to the results of better thermal safety of ultrasonic bone cutting than mechanical rotary instrumentation [1]. The clinically relevant differences in thermal safety margins are in the maximum temperature difference of 4.3° C at 1mm distance during continuous irrigation and a growth to 6.7° C during no irrigation. It has been experimentally proved that bone temperatures above 47C over a time that is longer than 60 seconds result in irreversible osteocyte necrosis and defective bone regeneration [2]. The fact that rotary instruments reached this threshold in 80 out of 100 cases without irrigation but piezosurgery did not reach this critical measurement in all cases highlights the greater safety profile of ultrasonic cutting [3]. The physics of reduced heat production during piezosurgery is based on the fundamental differences between cutting physics of rotary instruments and piezosurgery instruments [4]. Rotary burs cut bone with macroscopic friction with cutting edges hitting and scraping mineralized tissue and directly transforming mechanical energy to heat. Piezosurgery utilises high-frequency microvibrations (25-29 kHz) with little amplitude (60-200 µm) that cause microfractures by means of cavitation effects and propagation of mechanical stress instead of gross friction [5]. This micromechanical cut allocates energy on a bigger bone mass and time, impacting less heat concentration locally [6]. Cavitation phenomenon that involves the creation of microscopic vapor bubbles in irrigation fluid due to rapid ultrasonic oscillations, offers further cooling due to latent heat of vaporization and further circulation of the fluid around the edge of the cutting site [7]. This could be the reason why piezosurgery did not have higher temperatures despite lower irrigation than with rotary cutting that was well-irrigated. Ultrasonic vibrations create the acoustic streaming effect which actively pumps the fluid into the cutting site and it could be the reason of good temperature control [8]. The much smaller rate of temperature increase with piezosurgery (0.42° C/s compared to 1.24° C/s in continuous irrigation) gives further thermal safety by allowing all of the time to reach high critical temperatures. Biological tissue has thermal relaxation characteristics and the processes of heat dissipation by conduction and perfusion compete with the processes of heat generation [9]. Slow increase in temperature will enable thermal dissipation processes to reduce peak temperatures and decrease the likelihood of injury [10]. A possible clinical drawback is that piezosurgery is a relatively long procedure (47.3 vs 28.4 seconds to complete the same osteotomies) when compared to traditional osteotomies. Yet, our correlation test showed that this long duration did not significantly raise thermal elevation with piezosurgery (r=0.284, p=0.129) as compared to high degree of duration-temperature correlation of rotary instruments (r=0.742, p<0.001). This observation implies that the fear of thermal build up in the course of lengthy piezosurgery application might be unwarranted; the key thing is that there should be sufficient irrigation [11]. Spatial analysis of temperature showed that thermal gradients spread to a depth of 5mm surrounding the site of osteotomy, with rotary equipment sustaining very high temperature at all the distances measured. This observation has clinical implications to procedures that are close to the temperature sensitive areas such as tooth roots, inferior alveolar nerve, or the already placed implants [12]. The increased thermal gradient around rotary instrument cutting locations (3.10C/mm vs 2.40C/mm at 3-5mm distance) signifies more focal heat that can be hazardous to the surrounding tissues [13]. The absolutely vital role which irrigation played in both types of instruments was observed, but the degree of necessity seemed to be higher with rotary instruments. The mean temperature increase of 19.7° C without irrigation in rotary cutting as compared to 10.9° C in piezosurgery indicates that ultrasonic cutting is intrinsically thermally safe even when remote cooling is not optimal [14]. This can be beneficial in clinical practice where there is restricted access to irrigation or when full fluid contamination minimization is preferable [15]. Clinical consequences are improved safety in temperature sensitive surgeries such as peri-implant osteotomy, harvesting of bone to be used in grafting and surgery around the neurovascularity. Regular temperatures kept below the necrotic levels by piezosurgery might result in better bone healing, fewer complications and greater integration of the implant into the bone, in the field of implant dentistry. There are some crucial restrictions that should be mentioned. First, this test *in vitro* model employed the use of bovine bone devoid of blood perfusion which can yield physiological cooling in clinical practice. Nonetheless, lack of perfusion is a form of conservative testing because *in vivo* temperatures would be lower. Second, single-pass osteotomy is in contrast to clinical interventions in which there are many repetitive cuts or drill cycles that can amass thermal damage. Third, room-temperature irrigation (20° C) is not comparable to clinical practice as body-temperature saline can be considered more comfortable to patients. Fourth, single power settings and operational parameters were evaluated; various settings will provide various thermal profiles. Fifth, there was the use of new instruments; clinical wear could interfere with cutting efficiency and heat generation. These limitations can be resolved in future studies using *in vivo* animal experiments to match temperature measurements with histologic damage assessment of bone, testing several operational parameters such as power and tip design, discussing the accumulation of temperature during repetitive cutting cycles simulations of real surgical activities and follow-up studies.

## Conclusion:

Piezosurgery proves to be much less heat producing and better thermally controlled than the common rotary instruments when performing osteotomy. It is the most effective in keeping the bone temperatures at levels that do not lead to necrosis and hence safer bone preservation even in cases of lowering the irrigation. Thus, we show piezosurgery as the method of choice on minimizing thermal damage in the temperature-sensitive surgeries.
